# Non-Linear Dynamical Analysis of EEG Time Series Distinguishes Patients with Parkinson’s Disease from Healthy Individuals

**DOI:** 10.3389/fneur.2013.00200

**Published:** 2013-12-11

**Authors:** Claudia Lainscsek, Manuel E. Hernandez, Jonathan Weyhenmeyer, Terrence J. Sejnowski, Howard Poizner

**Affiliations:** ^1^Institute for Neural Computation, University of California San Diego, La Jolla, CA, USA; ^2^Computational Neurobiology Laboratory, Howard Hughes Medical Institute, Salk Institute for Biological Studies, La Jolla, CA, USA; ^3^School of Medicine, Indiana University, Indianapolis, IN, USA; ^4^Graduate Program in Neurosciences, University of California San Diego, La Jolla, CA, USA

**Keywords:** Parkinson’s disease, electroencephalography, dopamine-replacement, classification, non-linear dynamical analysis

## Abstract

The pathophysiology of Parkinson’s disease (PD) is known to involve altered patterns of neuronal firing and synchronization in cortical-basal ganglia circuits. One window into the nature of the aberrant temporal dynamics in the cerebral cortex of PD patients can come from analysis of the patients electroencephalography (EEG). Rather than using spectral-based methods, we used data models based on delay differential equations (DDE) as non-linear time-domain classification tools to analyze EEG recordings from PD patients on and off dopaminergic therapy and healthy individuals. Two sets of 50 1-s segments of 64-channel EEG activity were recorded from nine PD patients on and off medication and nine age-matched controls. The 64 EEG channels were grouped into 10 clusters covering frontal, central, parietal, and occipital brain regions for analysis. DDE models were fitted to individual trials, and model coefficients and error were used as features for classification. The best models were selected using repeated random sub-sampling validation and classification performance was measured using the area under the ROC curve *A*′. In a companion paper, we show that DDEs can uncover hidden dynamical structure from short segments of simulated time series of known dynamical systems in high noise regimes. Using the same method for finding the best models, we found here that even short segments of EEG data in PD patients and controls contained dynamical structure, and moreover, that PD patients exhibited a greater dynamic range than controls. DDE model output on the means from one set of 50 trials provided nearly complete separation of PD patients off medication from controls: across brain regions, the area under the receiver-operating characteristic curves, *A*′, varied from 0.95 to 1.0. For distinguishing PD patients on vs. off medication, classification performance *A*′ ranged from 0.86 to 1.0 across brain regions. Moreover, the generalizability of the model to the second set of 50 trials was excellent, with *A*′ ranging from 0.81 to 0.94 across brain regions for controls vs. PD off medication, and from 0.62 to 0.82 for PD on medication vs. off. Finally, model features significantly predicted individual patients’ motor severity, as assessed with standard clinical rating scales.

## 1 Introduction

Parkinson’s disease (PD) is a common and progressive neurological disorder that adversely affects the quality of life of up to six million people worldwide (1). Classical models of the pathophysiology of PD have emphasized the anatomical segregation of multiple looping structures linking frontal cortex and the basal ganglia, distinct pathways within the basal ganglia, and excessive firing rates of basal ganglia output nuclei that lead to excessive tonic inhibition of thalamus and cortex (2, 3). However, it recently has become clear that temporal patterning within these looped structures is of critical importance, and that the deficiency in dopamine in PD results in markedly abnormal patterns of timing and synchronization within basal ganglia-thalamic-cortical circuits (4–6). It also is becoming clear that these abnormal timing patterns contain non-linear features (7), that non-linear features of basal ganglia neuronal activity may be important for information coding (8), and that dopamine treatment in PD patients reduces abnormal non-linear interactions between rhythms in the local field potential oscillations in basal ganglia nuclei (9).

Although EEG recorded at the scalp reflects grossly summed currents, studies that have simultaneously recorded scalp EEG and local field potentials in the basal ganglia (subthalamic nucleus) in PD patients have found that scalp EEG reflects processing within functionally coupled circuits connecting distinct cortical areas and basal ganglia (10–12). These results have led to the general conclusion that “tuning to distinct frequencies may mark and segregate related processing, over and above any anatomical segregation of processing streams” (11). Thus, cortically generated EEG signals recorded at the scalp may be used as a marker for the nature of the processing within altered basal ganglia-thalamic-cortical circuits. Indeed, abnormalities in resting-state and movement-related oscillatory brain activity have been observed in the EEG of PD patients using both linear and non-linear time series methods (13–25).

We have previously used non-linear dynamical analyses to characterize and distinguish the motor behavior of PD patients from healthy controls (26). However, to our knowledge, non-linear methods have not yet been used to classify changes in brain activity in scalp EEG due to PD and to dopaminergic therapy (27, 28). The primary goal of this study is to use data models based on delay differential equations (DDE) as non-linear time-domain classification tools to distinguish EEG recordings from PD patients on and off dopaminergic therapy and healthy individuals. Given that DDEs can uncover hidden dynamical structure from short segments of simulated time series of known dynamical systems in high noise regimes, as shown in a companion paper (29), we also characterized changes in EEG dynamics in PD patients and age-matched healthy controls.

## 2 Materials and Methods

### 2.1 Participants

Nine PD patients (6 female) and nine age-matched healthy older adults (Controls, 4 female) participated (Mean ± SD age: PD patients, 62.8 ± 8.4 years; controls, 64.3 ± 7.9 years, *t*-test for means, *p* > 0.05). All patients had mild to moderate clinically typical PD (Hoehn and Yahr stages 2 and 3), and their motor disabilities were responsive to anti-Parkinsonian medications. No patient had marked resting tremor, action tremor, or dyskinesias. Moreover, no patient had dementia or major depression (screened with the Mini-Mental State Examination (30) and Beck Depression Inventory (31)). No participant had any neurological or psychiatric disease in addition to PD for the PD participants. All participants were right-handed (32) with normal or corrected to normal vision. Clinical characteristics of the PD patients are given in Table 1.

**Table 1:** **Clinical characteristics of Parkinson’s disease patients**.

Patient ID	Sex	Age (years)	Disease duration^a^ (years)	UPDRS^b^ (ON, OFF)	H&Y score^c^ (ON, OFF)	Medications
PD01	F	65	8	20, 39	2, 2	Carbidopa/levodopa, pramipexole
PD02	M	70	17	47, 52	3, 3	Carbidopa/levodopa, amantadine, selegiline
PD03	M	47	7	42, 58	3, 3	Ropinirole XL, selegiline, rasagiline
PD04	F	70	7	30, 38	2, 2	Rasagiline, pramipexole
PD05	F	68	3	22, 28	3, 2	Rasagiline, carbidopa/levodopa
PD06	F	69	9	31, 37	3, 3	Carbidopa/levodopa, amantadine, selegiline, pramipexole
PD07	F	66	4	36, 44	3, 3	Carbidopa/levodopa, pramipexole, rasagiline
PD08	M	58	12	37, 41	2, 3	Carbidopa/levodopa/entacapone; rasagiline
PD09	F	52	9	33, 43	3, 3	Carbidopa/levodopa, rasagiline, amantadine, ropinirole XL

*^a^Duration is years since first remembered Parkinsonian symptom*.

*^b^UPDRS: United Parkinson’s Disease Rating Scale, Motor Section (maximum score of 108). Higher scores indicate greater motor impairments*.

*^c^H&Y stage: Hoehn and Yahr stage (maximum score of 5). Higher stages indicate more severe disease*.

PD patients were tested on (PD ON) and off (PD OFF) their anti-Parkinsonian medications in counterbalanced order on separate days. For off medication testing, patients were tested in the morning before taking their first medications of the day and having not taken their anti-Parkinsonian medication for at least 12 h (33). Prior to each session’s testing, the Unified Parkinson’s Disease Rating Scale (UPDRS) was administered to provide a clinical measure of each patient’s motor severity. All participants signed the informed consent document approved by the human subjects Institutional Review Board of the University of California San Diego.

### 2.2 Data acquisition and task

Electroencephalographic (EEG) data were acquired with a 64-channel active electrode EEG system (BioSemi Inc., ActiveTwo, Amsterdam, Netherlands) consisting of a cap plus four EOG electrodes, temporal to both eyes and above and below the right eye, two EMG electrodes on the trapezius and right and left sternocleidomastoids, and two reference electrodes on the left and right mastoids. Data were recorded at 512 Hz, and referenced to the average of the mastoid electrodes. The positions of the EEG sensors on the head were digitized with a electromagnetic motion tracking system (Polhemus, FASTRAK, Colchester, VT, USA).

In this study, we analyzed EEG data from two sets of 50 randomly selected 1-s “baseline” trials, collected between trials as participants waited for a “go” cue to reach for and grasp a virtual rectangular object. During these resting intervals, subjects rested their right thumb and index finger on a virtual starting dock. Participants were provided haptic as well as visual feedback of the dock, so that they felt their hands resting on a solid surface using two haptic robots (Phantom Premium 1.0, Geomagic, Wilmington, MA, USA). Overall, a maximum of 360 (10 blocks of 36 trials) trials were performed by each participant, with rest provided between blocks to limit fatigue [see Ref. (34), for details of the experiment and behavioral results].

### 2.3 Data preprocessing

**Figure 1:**
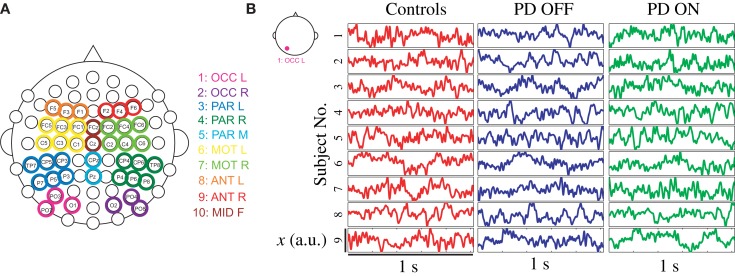
**(A)** Overview of EEG data clusters used in analysis, and **(B)** randomly selected EEG time series from each participant sampled at 512 Hz from left occipital cluster during 1-s baseline trial. Note: OCC, occipital; PAR, parietal; MOT, motor; ANT, anterior; L, left; R, right; F, frontal.

Raw EEG data were imported into EEGLAB using MATLAB (The MathWorks, Natick, MA, USA) for processing (35). Data were high-pass filtered at 1 Hz to remove drift and low-pass filtered at 55 Hz to remove line noise. EEG artifacts associated with eye and other muscle movement were removed using independent component analysis (ICA) (36). Based on the topography, spectra, and trial-to-trial characteristics of ICA components, good fit ICA components were selected and used to generate back-projected EEG data, which will be referred to as clean EEG. The 64 EEG channels were then grouped into 10 clusters covering frontal, central, parietal, and occipital brain regions for analysis (Figure 1A), using the mean voltage from 2 to 6 electrodes depending on the cluster. Figure 1B presents a representative EEG time series of the left occipital cluster from each participant and is illustrative of the variability in EEG seen across subjects.

### 2.4 Feature extraction

**Figure 2:**
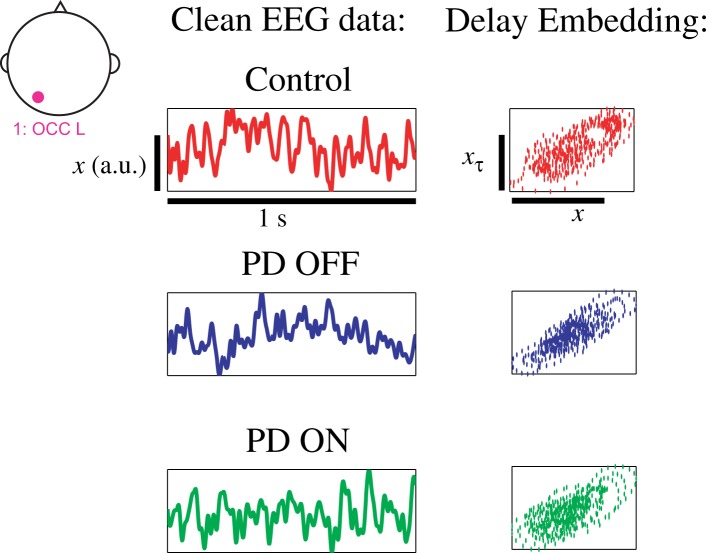
**EEG data from left occipital cluster of three exemplary participants with phase portrait reconstructed from left occipital cluster, using a delay τ of 8 ms**.

Delay differential equations (DDE) were used as a generic non-uniform embedding tool to extract feature vectors from clean EEG data for classification. The method introduced here is based on non-linear modeling methods known to be effective in classifying short time series (26, 29, 37–39). Typical phase portraits for a PD patient on and off medications and healthy control subjects are shown in Figure 2. The phase portraits suggest that delay embeddings may be able to uncover underlying dynamical structure of EEG data in all participants.

Lainscsek et al. (26) proposed a genetic algorithm to find a single DDE model for the classification of PD movement data. Similar to Ref. (40), we propose an exhaustive search of models and delays to find the model and delay combination that can best separate EEG data from PD patients on and off dopaminergic therapy and healthy individuals. Candidate polynomial DDE models include

(1) $$\begin{array}{ccc} ẋ & =a_{1}\;x_{\tau_{1}}+a_{2}\;x_{\tau_{2}}+ & \\ & \;a_{3}\;x_{\tau_{1}}^{2}+a_{4}\;x_{\tau_{1}}\;x_{\tau_{2}}+a_{5}\;x_{\tau_{2}}^{2}+\\ & \;a_{6}\;x_{\tau_{1}}^{3}+a_{7}\;x_{\tau_{1}}^{2}\;x_{\tau_{2}}+a_{8}\;x_{\tau_{1}}\;x_{\tau_{2}}^{2}+a_{9}\;x_{\tau_{2}}^{3} \end{array}$$

where *x* = *x*(*t*) and $x_{\tau_{j}}=x\left(t-\tau_{j}\right),$ with some of the *a_i_* equal to zero [see structure selection in the companion paper (29)]. In this study, we only considered models with two to three terms, a maximum of two delays, and terms with up to cubic order of non-linearity, so as to limit computational demands. In total we have 18 two-term models and 32 three-term models [see companion paper (29) for a full list of models]. We had one linear model and all others were non-linear.

DDE models were fitted to individual 1-s trials of clean EEG data, and model coefficients and least square error were used as features for classification. Supervised structure selection was performed in order to identify the DDE model form and delays that best classified two classes of data. In total three classifiers were considered: (1) Control vs. PD OFF, (2) PD ON vs. OFF, and (3) Control vs. PD ON. Training data consisted of 50 randomly selected trials from each of the 10 EEG data clusters from each participant. For each classifier, training was first carried out on data from 6 randomly selected participants from each group on a total of 600 trials, and then testing was performed on data from the 3 remaining participants from both groups (300 trials) using repeated random sub-sampling validation [Ref. (41), see companion paper (29)]. For each trial, the DDE features (i.e., coefficients and least square error) of all 50 DDE models with delays between 1 and 50 time steps were computed and the linear separating hyperplane between groups in each classifier was found, which consisted of a linear set of weights for each DDE feature estimated via a singular value decomposition (SVD) algorithm. Testing consisted of using the previously calculated weights to compute the separating hyperplane on untested data. For each trial and model and delay combination, classification performance was then measured using the area under the receiver operator characteristic (ROC) curve *A*′. This process was repeated 84 times with different training and testing subjects, with each subject used equally for both training and testing. A combination of the 30 best performing model and delay combinations were then used to extract a mean set of weights that was used for testing on a new set of data from each participant, which consisted of an additional 50 randomly selected trials. For details on the methodology, see Ref. (29).

### 2.5 Statistical analysis

Linear mixed models were used to evaluate the effect of cluster location and classifier (Control vs. PD OFF, PD ON vs. OFF, and Control vs. PD ON) on the classification performance in both original and new EEG data. As fixed effects, we entered cluster location and classifier along with their interaction and the training and testing combination. As random effects, we had intercepts for cluster, as well as by-cluster and by-training and testing combination random slopes for the effect of classifier. In addition, linear mixed models were used to examine the effect of cluster location and PD (Control vs. PD OFF) or medication (PD ON vs. OFF) on the mean logarithmic power at theta (4–8 Hz), alpha (8–12 Hz), and beta (12–30 Hz) frequency bands. As random effects, we included the subject, cluster, cohort as well as subject by-cluster and cohort interaction. *P*-values were obtained by likelihood ratio tests of the full model with and without the effect in question. A significance level of *p* = 0. 05 was used to test for statistical significance. To control for multiple comparisons, Hochberg’s step-up method was used in this secondary analysis. Pearson correlations (42) between the DDE model distance from the dividing hyperplane and severity of motor impairment in PD patients tested the degree of relationship between model output and PD motor impairment. All statistical analyses were done using R version 3.0.1 (43); linear mixed models were fit using lme4 version 0.999999-2 (44).

## 3 Results

**Figure 3:**
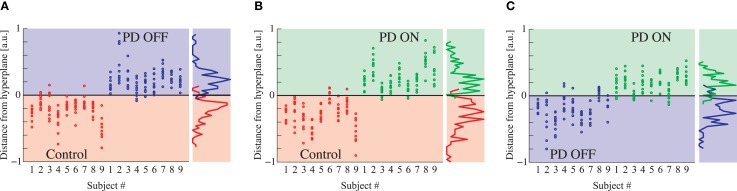
**(A)** DDE model output distance from the hyperplane in classification of controls vs. PD patients off medications, **(B)** controls vs. PD patients on medications, and **(C)** PD patients on vs. off medications. Each column represents all 10 cluster classification outputs for a given subject.

Even brief 1-s segments of EEG data in PD patients and healthy age-matched controls demonstrate dynamical structure, as seen by differences in the distance to the hyperplane in all groups (Figure 3). Given that changes in the distance from the hyperplane reflect changes in the underlying dynamics of the system (29), the distinct separation of both PD patients on and off medication vs. controls (Figures 3A,B) are indicative of distinct and quantifiable changes in EEG dynamics due to PD, irrespective of treatment. The separation between PD patients on and off medication is relatively strong but less than that of the other two comparisons (Figure 3C), indicating increased similarities in EEG dynamics (Figure 3C).

**Figure 4:**
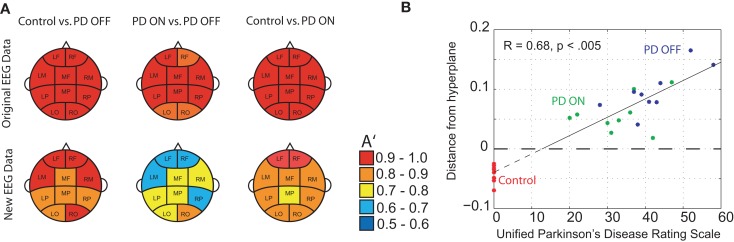
**(A)** Classification performance of three different classifications using *A*′ (area under the receiver-operating characteristic curve) on the mean of the original 50 randomly selected 1-s trials of baseline EEG used for training and the mean of 50 new 1-s trials from the same subjects, and **(B)** relationship between DDE model output’s distance from a hyperplane, and severity of motor impairment in PD patients, as evaluated by the Unified Parkinson’s Disease Rating Scale (UPDRS). Controls are included to serve as a reference.

DDE model output on the means from one set of 50 trials provided nearly complete separation of PD patients off medication from controls: across brain regions, the area under the receiver-operating characteristic curves, *A*′, varied from 0.95 to 1.0. For distinguishing PD patients on vs. off medication, classification performance *A*′ ranged from 0.86 to 1.0 across brain regions (Figure 4A). Lastly, for distinguishing PD patients on medication from controls, classification performance *A*′ varied from 0.97 to 1.0. Moreover, the generalizability of the model to the second set of 50 trials was excellent, with *A*′ ranging from 0.81 to 0.94 across brain regions for controls vs. PD off medication, from 0.62 to 0.82 for PD on medication vs. off, and from 0.74 to 0.92 for controls vs. PD on medication (Figure 4A). Finally, model features significantly predicted individual patients’ motor severity, as assessed with standard clinical rating scales (Figure 4B, Pearson correlation coefficient *R* = 0.68, *p* < 0.005).

Overall, cluster location was found to significantly affect classification performance in both the original [*χ*^2^(9) = 18.9, *p* = 0.026] and new [*χ*^2^(9) = 26.1, *p* = 0.002] EEG data. Classification performance, as evaluated by *A*′, was affected by the classifier (Control vs. PD OFF, PD ON vs. OFF, and Control vs. PD ON) on new data only [*χ*^2^(2) = 16.1, *p* < 0.001]. Specifically, overall classification performance of PD patients on medication vs. off decreased by 0.165 relative to the classification of PD patients off medication from controls (*A*′ = 0.886). In addition, the interaction between cluster location and classifier was found to affect *A*′ in both original [*χ*^2^(18) = 85.9, *p* < 0.001] and new [*χ*^2^(18) = 77.5, *p* < 0.001] trials. Relative to Control vs. PD OFF classification performance at the left occipital cluster (OCC L), *A*′ increased at the left motor cluster (MOT L) by 0.023 ± 0.007 (standard error) in original trials and by 0.097 ± 0.017 in new trials. PD ON vs. OFF classification performance was best in the mid frontal cluster (MID F) in original trials and in the right occipital (OCC R) cluster in new trials, relative to OCC L. Control vs. PD ON classification performance was best in the right frontal cluster (ANT R) in new trials (i.e., 0.063 ± 0.016 increase, relative to OCC L).

**Figure 5:**
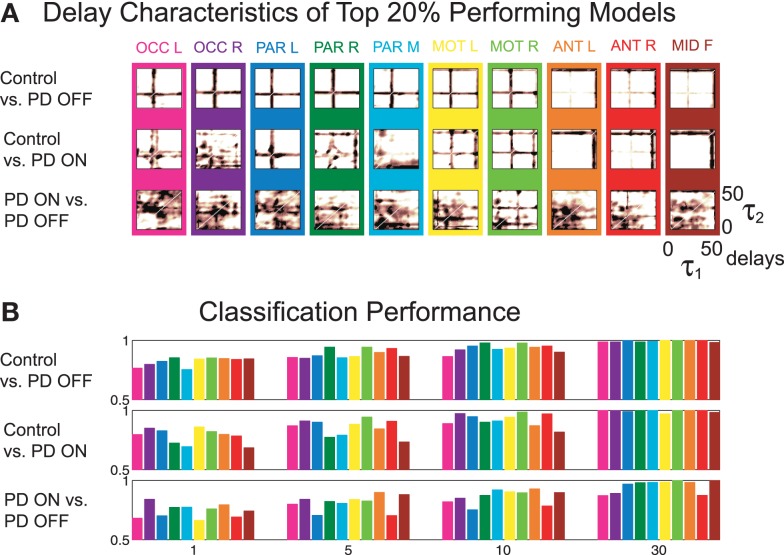
**(A)** Time delay characteristics of top 20% performing models, and **(B)** classification performance of three different classifications using *A*′ on the mean of the original 50 1-s trials, using 1, 5, 10, and 30 combined models.

**Figure 6:**
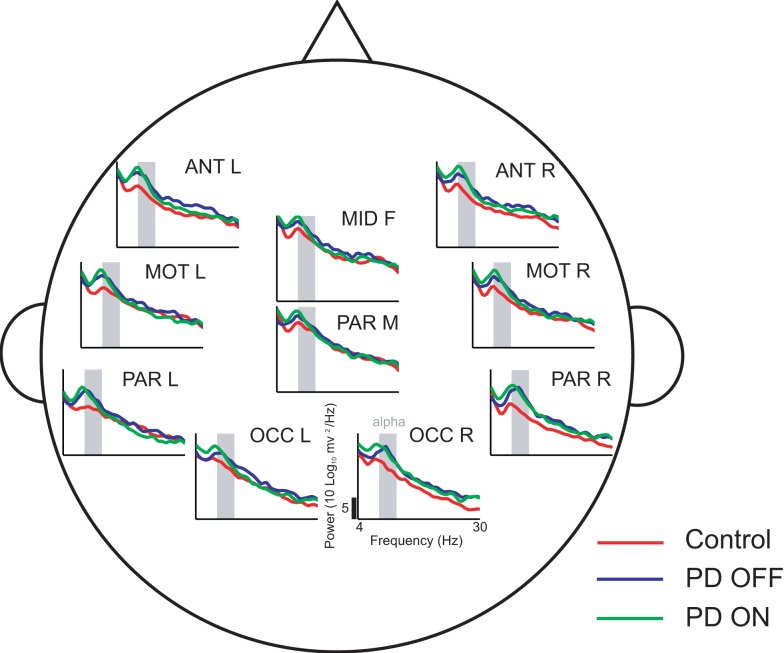
**Power spectral density estimates across all ten clusters using 50 1-s trials**. In comparison to controls, PD patients off medications demonstrate increased theta activity at a few cluster locations, such as the anterior clusters (ANT L and ANT R)

**Figure 7:**
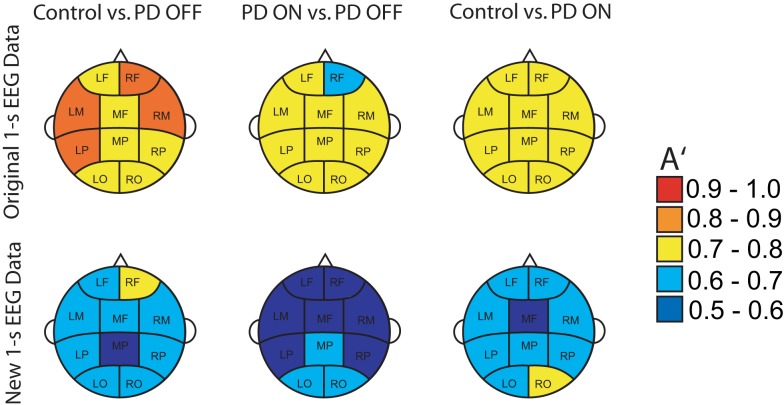
**Classification performance of three different classifications using *A*′ on all the original 1-s trials of EEG used for training and new 1-s data trials from the same subjects**.

The delay characteristics of the top performing DDE models indicate that to best differentiate between PD patients and controls, longer time delays (i.e., time delays closer to 50 time steps or 100 ms) are needed in frontal clusters (i.e., anterior left, anterior right, and mid frontal), in comparison to all other clusters (Figure 5A). In contrast, differentiation of PD patients on vs. off medications leads to a more diffuse selection of time delays, with shorter time delays used in the posterior cortices. Moreover, the diffused selection of time delays is indicative of a greater dynamic range in PD patients vs. controls. Considering each cluster, we demonstrate the effect of an increased number of combined models on the classification performance (Figure 5B). Based on traditional spectral analysis output, no statistically significant differences in mean power spectra were observed between PD patients and controls nor between PD patients on and off medications [*p* > 0.0042 (i.e., *p* = 0.05/12)]. However, a significant interaction between PD patients off medication vs. control and cluster location was identified in the theta [*χ*^2^(9) = 25.9, *p* = 0.002] frequency band (Figure 6). Moreover, dopaminergic therapy demonstrated a significant interaction with cluster location in the theta [*χ*^2^(9) = 34.6, *p* < 0.001] and beta [*χ*^2^(9) = 24.9, *p* = 0.003] frequency bands, consistent with the literature (45). Given, these differences in spectral power density in PD patients vs. controls, and due to the observed effects of dopaminergic therapy, we examined how well our non-linear methods could classify subjects based on each individual 1-s trial (Figure 7). Using a single second of data, allowed us to differentiate between PD patients off medication and controls (*A*′ = 0.73 − 0.80), PD patients on and off medication (*A*′ = 0.74 − 0.82), and PD patients on medications and controls (*A*′ = 0.69 − 0.76) with a classification performance well above chance.

## 4 Discussion and Conclusion

We have shown that differences in EEG dynamics due to PD and to dopaminergic therapy are detectable using DDE models. Moreover, we found that DDE model output of EEG significantly correlated with the patient’s motor impairment severity (Figure 4B). This finding is consistent with prior work showing that DDE model output of movement time series likewise correlated with PD severity (26). Given the excellent classification performance of PD patients on and off medication vs. controls, DDEs may provide a promising non-linear time-domain classification tool for objectively and automatically measuring changes in neural dynamics due to a broad range of neurological disorders, and might eventually serve as a biomarker, or one component of a biomarker, for PD. This work is consistent with recent advances in the study of sensory-motor circuitry that have demonstrated non-linear temporal dynamic irregularities in motor-related neuronal activity due to Parkinson’s disease (46). These irregularities have also been shown to be amenable to dopaminergic treatment (7, 47, 48).

This study provides further evidence that non-invasive, scalp EEG analysis can be used to detect abnormalities in the function of basal ganglia-cortical circuits in PD patients (4, 5). In particular, frontal clusters demonstrated excellent, generalizable, classification performance between PD patients and controls (Figure 4A). Consistent with findings of increased theta power in frontal areas due to PD (49), our traditional spectral analysis suggests differential changes in theta frequency power spectra as a potential explanation for this improved classification performance. Classification performance when distinguishing PD patients on vs. off medications was best in occipital or anterior clusters (Figure 4A), which coincides with reported decreases in frontal theta and occipital beta due to dopaminergic therapy during rest (45). We also found that PD patients off medication showed increased theta power in certain clusters relative to control subjects. However, we were unable to find significant differences due to either PD or dopaminergic therapy in other frequency bands, including beta. Lack of differences in beta power between PD patients and controls was an unexpected result. However, the short, 1 s EEG intervals used in the present study may not have provided a sufficient amount of time for beta activity to recover, and hence, may explain the similar power spectral density plots between PD patients and controls (Figure 6) described in the paper. It should be noted that power spectra is a linear feature of EEG data. Thus, the excellent EEG classification performance using DDE models are likely relying on non-linear features of EEG data, which warrant further investigation.

Based on non-linear dynamical classification of short time series of the Rössler system in a companion paper (29), we found that the distance to the hyperplane is related to the underlying dynamic parameters of the system. Given the distinct separation in PD patients versus controls based on the distance to the hyperplane, this study further affirms that PD results in changes in non-linear temporal brain dynamics (19, 28, 46) and demonstrates such changes in EEG. The increased difficulty in distinguishing PD patients on versus off medication, particularly when generalized to new data, suggests that both groups have more overlapping EEG dynamics. Together with the broader distribution of time delays selected in the top performing models in classifying PD patients on and off medication, these findings are consistent with observed increases in the local entropy of EEG due to PD (19, 23), as higher entropies correspond to decreased predictability of EEG dynamics.

Interestingly, the increased dynamic range observed in the EEG data of PD patients in this study (Figure 4B) is suggestive of increased complexity in cortical activity, consistent with findings of higher dimensional EEG signals in PD patients, when compared to healthy controls during imagined or actual movements (18), and during sleep (50). However, both animal and human studies have demonstrated pathologically increased oscillatory synchronization in the basal ganglia due to Parkinsonism (51–54), which could be indicative of a reduction in signal complexity (55). Furthermore, recent findings suggest a strong negative correlation between signal complexity and some dimensions of motor impairment in PD patients, based on subthalamic nucleus local field potentials recorded at a resting state (56). Further studies are necessary to resolve these discrepancies and further examine this important issue.

In conclusion, this study provides a unique and novel time series method for analyzing the dynamics of neural activity in patients with Parkinson’s disease. Given the excellent classification performance observed in DDE models when distinguishing brief and sparse 1-s EEG recordings from PD patients on and off dopaminergic therapy and healthy individuals, this study provides a crucial first step toward the development of an objective and automatic method of tracking the progression of PD using EEG time series. Additional training on a larger pool of PD patients and healthy age-matched controls, and expansion to longer time delays, should allow for a further refinement of the relevant dynamical features for classification of PD. As this study was carried out on a limited sample of subjects, additional subjects are needed for a prospective test of this classification method to evaluate the generalizability of our findings. Furthermore, further study of the non-linear dynamics of cortical activity and comparing with intra-cortical data collected during surgery should provide a better understanding of the underlying pathophysiology of the basal ganglia-thalamic-cortical system in Parkinson’s disease.

## Conflict of Interest Statement

The authors declare that the research was conducted in the absence of any commercial or financial relationships that could be construed as a potential conflict of interest.
